# An integrated analysis to predict micro‐RNAs targeting both stemness and metastasis in breast cancer stem cells

**DOI:** 10.1111/jcmm.14090

**Published:** 2019-02-01

**Authors:** Mahsa Rahimi, Ali Sharifi‐Zarchi, Javad Firouzi, Mahnaz Azimi, Nosratollah Zarghami, Effat Alizadeh, Marzieh Ebrahimi

**Affiliations:** ^1^ Department of Medical Biotechnology, Faculty of Advanced Medical Sciences Tabriz University of Medical Sciences Tabriz Iran; ^2^ Department of Stem Cells & Developmental Biology at Cell Science Research Center, Royan Institute for Stem Cell Biology & Technology ACECR Tehran Iran; ^3^ Department of Computer Engineering Sharif University of Technology Tehran Iran; ^4^ The Umbilical Cord Stem Cell Research Center Tabriz University of Medical Sciences Tabriz Iran

**Keywords:** breast cancer stem cell, EMT, metastasis, miRNAs, self‐renewal

## Abstract

Several evidences support the idea that a small population of tumour cells representing self‐renewal potential are involved in initiation, maintenance, metastasis, and outcomes of cancer therapy. Elucidation of microRNAs/genes regulatory networks activated in cancer stem cells (CSCs) is necessary for the identification of new targets for cancer therapy. The aim of the present study was to predict the miRNAs pattern, which can target both metastasis and self‐renewal pathways using integration of literature and data mining. For this purpose, mammospheres derived from MCF‐7, MDA‐MB231, and MDA‐MB468 were used as breast CSCs model. They had higher migration, invasion, and colony formation potential, with increasing in stemness‐ and EMT‐related genes expression. Our results determined that miR‐204, ‐200c, ‐34a, and ‐10b contemporarily could target both self‐renewal and EMT pathways. This core regulatory of miRNAs could increase the survival rate of breast invasive carcinoma via up‐regulation of *OCT4, SOX2, KLF4, c‐MYC, NOTCH1, SNAI1, ZEB1, *and *CDH2 *and down‐regulation of *CDH1*. The majority of those target genes were involved in the regulation of pluripotency, MAPK, WNT, Hedgehog, p53, and transforming growth factor β pathways. Hence, this study provides novel insights for targeting core regulatory of miRNAs in breast CSCs to target both self‐renewal and metastasis potential and eradication of breast cancer.

## 1 INTRODUCTION

Effective treatment of breast cancer is faced to a number of hurdles including resistance to therapies, metastasis, and recurrence.^1^ There are intensified evidences regarding heterogeneity in breast cancer cell population, which originates from a very small subset of cells named cancer stem cells (CSCs).^2^ CSCs have self‐renewal ability and are responsible for initiating tumourigenesis in immunodeficient models,^3^ maintenance, and clinical outcomes of treatments.^4^

Although CSCs are very important from clinical points of view, the molecular mechanisms and pathways, which are active in them, are not fully identified. Recent progress has highlighted the significant role of miRNAs in controlling the stemness and metastasis in CSCs. In this way, several miRNAs were known to be differentially expressed in CSCs or normal stem cells, also their role has been studied in targeting genes and networks involved in stemness properties,^5^ cell proliferation, and differentiation.^6^, ^7^, ^8^, ^9^

A relationship between epithelial to mesenchymal transition (EMT) and self‐renewal and mammosphere formation capacity has recently defined with ectopic expression of *TWIST* or *SNAI* in human mammary epithelial cells.^10^ Consistently, mammosphere‐forming activity is abrogated in breast CSCs after the EMT is shut down.^11^ Alignment of EMT with the CSCs signature was also found in cells derived from a breast cancer lung metastasis.^12^ More importantly, many signalling pathways, such as Wnt, Notch, and Hedgehog, that regulate EMT also drive self‐renewal.^13^, ^14^, ^15^ Based on our knowledge, identifying potential regulatory miRNAs responsible for self‐renewal and EMT controlling could facilitate the detection of metastatic cell with the ability of seeding and enabling the discovery of therapeutic targets. Here, we presented an integrative experimental and computational approach for identifying miRNAs probably responsible for of CSCs potential and metastasis.

## 2 MATERIALS AND METHODS

### 2.1 Bioinformatics and computational analysis

First, we performed a systematic literature review on Pubmed and Coremine website to identify all related articles to our study with keywords: “Human breast cancer cell lines, CSC, self‐renewal, stemness, microRNA, metastasis, and EMT.” Briefly, we also looked for both miRNA and mRNA expression profiles on NCBI GEO database by searching the same keywords. Consequently, after the literature mining, studies with incomplete data were excluded from the analysis if (i) the review articles or letters, (ii) studies with insufficient or inaccessible data, and (iii) studies that are not related to CSCs and homo sapiens. After full text reviewing, all the miRNAs reported in each study were compiled in a list, and then, the most frequent miRNAs regulate the stemness and metastasis genes were highlighted. The targets of the miRNAs were predicted using TargetScan^16^ and miRWalk.^17^, ^18^ Each miRNA list with their target genes was reviewed. As the most of miRNAs at least connected to two genes in metastasis list and to three genes in stemness list, therefore, we selected common miRNAs regulating at least three stemness and two metastasis genes (Figure S1). Subsequently, we computed the differential expression fold changes and *P*‐values (using two‐sided Student’s *t* test) between mammospheres vs adherent culture (at least two fold‐change differential expression, *P* < 0.05).

Enrichr^19^, ^20^ on KEGG pathways was used to identify pathways that were affected by the target genes of each miRNAs. We also performed GO functional enrichment analysis (biological process, molecular function, and cellular component) by the same tool. The cut‐off criterion was *P* < 0.05. In addition, network analysis of miRNA targets was constructed to visualize the interaction between miRNAs and their target genes that were integrated and mapped in a network structure using miRTargetLink Human.^21^

### 2.2 Cell line and monolayer culture

Human breast epithelial adenocarcinoma cell lines (MCF‐7, MDA‐MB231, MDA‐MB468) were purchased from Iranian Biological Resource Center (IBRC). They were cultured in DMEM—Dulbecco’s Modified Eagle Medium (GIBCO,USA) supplemented with 10% heat inactivated foetal bovine serum (FBS; Invitrogen), 1% non‐essential amino acid, 2 mmol/L L‐glutamine, and 1% penicillin/streptomycin at 37**°**C in a humidified atmosphere with 5% CO_2_.

### 2.3 Generation of mammosphere cultures

To form mammospheres, we prepared two types of non‐adherent plates. In one experimental group, the standard tissue culture plates were covered with 1.2% poly 2‐hydroxyethyl methacrylate (p‐HEMA) (Sigma), and in the other group, plates were covered with 1% agar to prevent the cells attachment. Subsequently, 2 × 10^4^ cells of single cells in serum‐free medium consisted of DMEM and supplemented with 20 ng/mL epidermal growth factor (Royan Institute, Iran), 20 ng/mL basic fibroblast growth factor (Royan Institute), 2% B27 (no vitamin A; GIBCO, USA), and 2 mmol/L L‐Glutamine. The media were refreshed every 48 hours (without removing the old media), and finally, the mammospheres were formed at 37°C under a 5% humidified CO_2_ atmosphere after 7 days.

### 2.4 Mammosphere‐ and colony‐forming efficiency assay

Mammosphere‐forming efficiency was calculated by dividing the number of mammospheres, which are greater than 60 μm in number of seeding cells. All experiments were performed in each generation of mammospheres in triplicates.

To compare the colony‐forming capacity of adherent cells and mammospheres, 200 cells of each group were counted and replated in a complete medium containing DMEM supplemented with 10% FBS, 1% non‐essential amino acids, 2 mmol/L L‐glutamine, and 1% penicillin/streptomycin in six‐well plates. After 10 days, cell colonies were fixed with 4% paraformaldehyde and stained with 0.05% crystal violet (Sigma) and the round shape colonies with more than 400 μm diameter were counted using an inverted microscope (Tokyo, Japan Microscope brand).

### 2.5 Cell invasion and migration assay

Adherent cells and mammospheres of luminal phenotype (MCF‐7) and triple negative basal phenotype (MDA‐MB231 and MDA‐MB468) were grown to 80% confluence; then, adherent cells were starved in serum‐free medium the day before the assay. The next day, 1 × 10^5^ cells seeded onto the top chambers of transwell inserts of 8‐μm pore size filter (BD, USA) coated with 0.5 mg/mL matrigel (BD, USA) in a six‐well plate. At the bottom of the chambers, DMEM containing 10% of FBS was added, and the cells were then cultured for 10 hours at 37°C in a 5% humidified CO_2_ incubator. Finally, cells on the top surface of the filter were removed by using a cotton swab. Cells on the bottom of the filter were then fixed with 4% paraformaldehyde for 30 minutes and stained with 0.05% crystal violet. The chambers were then washed in PBS, counted using an inverted microscope with either a 4× or a 10× objective lens using cell science software and plotted as the percentage of invading of the total number of plated cells. For cell migration assay, all steps were carried out similar to those in the invasion assay except for the matrigel coating. The experiments were performed in triplicates.

### 2.6 RNA isolation and cDNA synthesis

Total RNAs with retention of small RNAs were extracted from the adherent cells (as control groups) and mammospheres (as experimental groups) using TRIzol reagent (Qiagen) according to the manufacturer’s instructions. The concentration and purity of extracted RNA were determined by UV absorbance at 260 and 280 nm (260/280 nm) in spectrophotometer. The integrity of RNA samples was checked by gel electrophoresis. Two micrograms of total RNA was used to generate cDNA using cDNA synthesis kit (TaKaRa, Japan) according to the manufacturer’s instructions.

### 2.7 Real‐time reverse transcriptase PCR

The expression level of stemness‐ and metastasis‐related genes was evaluated using quantitative real‐time reverse transcriptase PCR (RT‐PCR). Ten microlitres of reactions containing 2.5 μL of SYBR Green PCR mix (TaKaRa, Japan) and 1 μL of each primer with 5 pmol/μL concentration was subjected for QRT‐PCR using Applied Biosystems Instrument (ABI) (Thermo Fisher) with specific primers including stemness‐related genes (*OCT4, SOX2, NANOG, KLF4, NOTCH, c‐MYC*, and *CD133*) and metastasis‐related genes (*CDH1, CDH2, SNAIL1, SNAIL2, TWIST1, TWIST2*, and *ZEB1*) (Table S1). Expressions of these genes were normalized according to the expression of *β‐ACTIN*. The PCR thermal profile included 95°C for 10 minutes, 40 cycles of denaturation at 95°C for 10 seconds, annealing at 60°C for 20 seconds, and elongation at 72°C for 20 seconds. A final melting curve analysis from 65 to 95°C was performed and the relative level was analysed using the 2^−ΔΔCT^ values.

### 2.8 MiRNAs validation by real‐time PCR

MiRNAs were evaluated by performing SYBR green qRT‐PCR. In brief, 100 ng of total RNA containing the miRNAs was poly adenylated by poly (A) polymerase and reverse transcribed to cDNA using RT enzyme. First‐strand cDNA synthesis reaction was provided in the PARSGENOME MiR‐Amp kit according to the manufacturer’s instructions. Each reaction was performed in a final volume of 10 μL containing diluted cDNA and PCR master mix and all reactions were run in triplicates. The qRT‐PCR reaction was performed using Applied Bio systems real‐time PCR Instruments (ABI) according to the manufacturer’s protocol. The expression levels of miRNAs were normalized against internal controls U6 primer as a housekeeping gene control.

### 2.9 miRNAs vs pathways heat maps

The DIANA‐miRPath v3.0 was applied to create advanced visualizations of miRNAs contributed in self‐renewal, EMT, and both self‐renewal and EMT vs pathways heat maps. Heat maps are graphical representations of data where values in a matrix are represented as colours.^22^ This web server uses the hierarchical clustering results of pathways and miRNAs on separate axes, in order to make the heat map visualization. These visualizations enabled us to identify patterns, which were not simply distinct their relationships and levels of interaction. “Significance Heat Maps” and “Targeted Pathways Heat Maps” are two options for heat map calculation, in the case of cluster analysis. Therefore, numerous miRNA‐miRNA, miRNA‐pathway, and pathway‐pathway relationships were identified by using this tool.

### 2.10 Survival analysis and definition of miRNA‐related prognostic signature

For assessment of overall survival implications for significant microRNAs, the PROGmiR tool was used as publicly available data sets.^23^ The breast cancer expression data were included the Cancer Genome Atlas dataset (https://cancergenome.nih.gov) and included 841 cases of breast invasive carcinoma (BRCA).

### 2.11 Statistical analysis

In vitro characterization of mammospheres derived from MCF‐7, MDA‐MB231, and MDA‐MB468 cells is presented as the mean ± SD of at least three different experiments. Two‐tailed Student’s *t* test and analysis of variance (ANOVA) were performed to evaluate the difference between the mean values. To detect the correlation of miRNA and mRNA expression levels, Spearman’s rank correlation test was used. For this, each group was done at three independent replicate and each replicate was done as duplicate. A two‐tailed with *P* < 0.05 was considered statistically significant for all experiments. For functional enrichment analysis, target genes of selected miRNAs were submitted to Enrichr database.

## 3 RESULTS

### 3.1 Agar‐coated plate and DMEM as culture media enhance sphere formation ability in breast cancer stem cells

**Figure 1:**
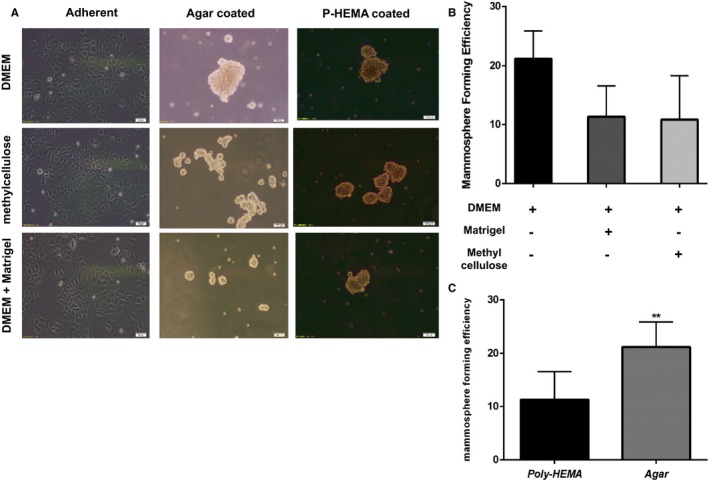
Cell morphology and efficiency of mammospheres derived from MCF‐7 cultured in different media and coating layers. To optimize the culture medium for mammosphere culture, DMEM alone or in mixture of methylcellulose and matrigel was used in different groups. Moreover, p‐HEMA and Agar were used as coating layer to reduce cell attachment. (A) Compact mammospheres 10 days post culture. Scale bar represents 100 μm for 40× magnifications. (B) The mammospheres forming efficiency was higher in DMEM medium and (C) when plates were coated by agar. **P < 0.01

To find the best coating layer for providing low attachment surface for mammospheres formation of MCF‐7 cells, six‐well plates were coated with 1% agar and 1.2% poly‐HEMA, respectively (described in Section 2). Moreover, DMEM alone, mixture of DMEM with methylcellulose (1.2%), and mixture of DMEM with matrigel (25 mg/mL) were used as culture media to find the best medium for mammosphere culture. As shown in Figure 1, MCF‐7 cells in monolayer culture had epithelioid morphology, polygonal shape with the define boundaries between the cells. In mammosphere cultures, all groups formed compact spheres and were not dissociated easily by pipetting (Figure 1A), and they reached 1500‐1900 μm in diameter on the fourth day. Meanwhile, the mammospheres in agar‐coated plates and in the presence of DMEM medium were significantly larger in size, and their sphere efficiency was about two folds more than other tested groups (*P < *0.0001, Figure 1B,C).

### 3.2 Mammospheres revealed higher rate of self‐renewal and invasion compared to their parental cells

**Figure 2:**
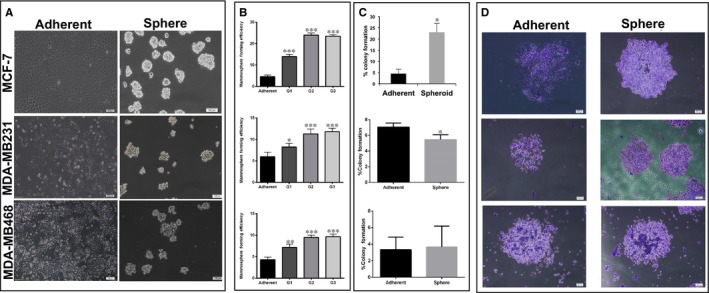
The sphere and colony formation ability of mammospheres derived from different breast cancer cell lines. (A) Morphology of mammospheres derived from MCF‐7, MDA‐MB231, and MDA‐MB468 cultured with DMEM and in agar‐coated plates. MCF‐7 formed the round and compact spheres, but other cell lines formed grape‐like spheres and looser over passages. (B) Mammosphere‐forming efficiency (MFE) based on the mean percentages of the number of spheres relative to the initial cell seeding number (means ± SD, N = 3). The sphere‐forming ability of mammospheres enhanced with increasing the passages. Bar indicated mean ± SD at least three different biological replicate. G indicated generation. (C) Colony number showed a significant increase under 3D culture conditions compare to adherent culture. The clonogenic ability of mammospheres was higher in MCF‐7‐spheroids (means ± SD, N = 3). (D) Morphology of colonies in mammospheres was mostly holoclones with define border and dense cellularity in all groups. *P < 0.05; **P < 0.01; ***P < 0.001

Three different cell lines (MCF‐7, MDA‐MB231 and MDA‐MB468) were cultured on agar‐coated palate and in the presence of DMEM to form mammospheres. All cells formed mammospheres. However, MDA‐MB231 and MDA‐MB468 formed loose and grape shape spheres compared to MCF‐7 that formed compact and dense mammospheres (Figure 2A). All mammospheres could be passaged continuously with significant increasing in the spheres formation ability (Figure 2B). All mammospheres were dissociated and subjected to colony formation assay in 2D and 3D models. The central part of each colony consisted of several layers of undifferentiated cells, whereas marginal part of each colony consisted of spindle and differentiated cells. Mammospheres derived from MCF‐7 were highly clonogenic; however, the MDA‐MB231‐mammospheres had lower clonogenic ability compared to adherent cells (Figure 2C). There were no differences in clonogenic ability of mammospheres derived from MDA‐MB468 and their adherent cells (Figure 2C). Morphologically, colonies in mammospheres were compact and large that is a characterization of holoclones (Figure 2D).

**Figure 3:**
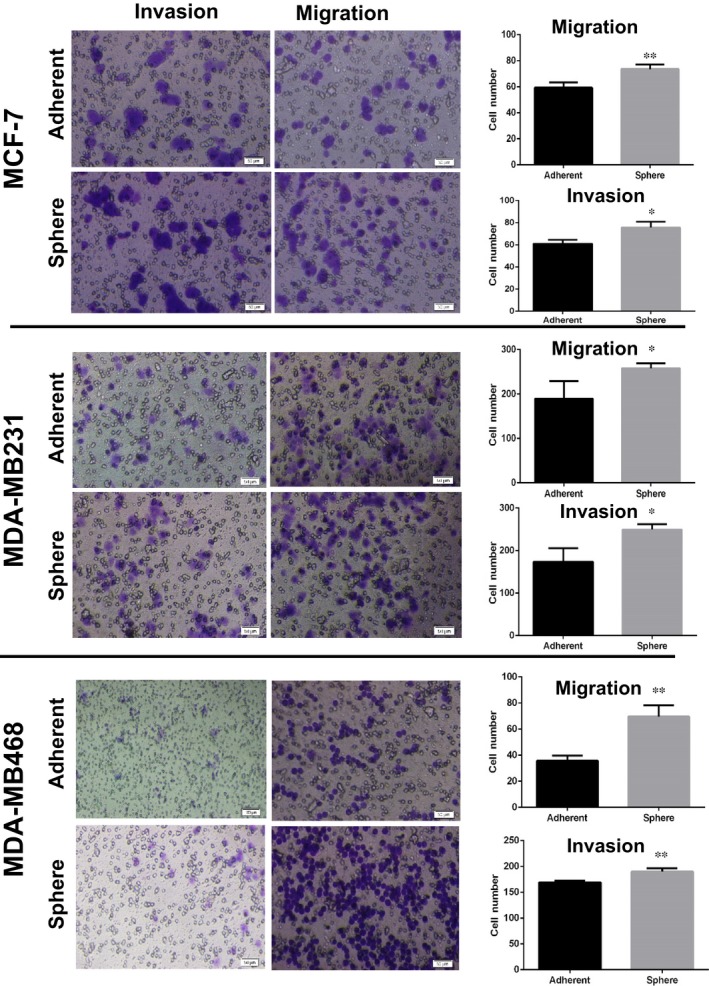
Cell migration and cell invasion of mammospheres compare to the parental cells. (Left) Cells were seeded at 100 000 cells per insert of a six‐well plate and allowed to migrate towards serum‐present medium for 10 h. Migratory cells on the bottom of the insert membrane were then fixed in formaldehyde and stained with crystal violet migrated and invaded cells that passed through 8 μm filters with/without matrigel. Magnification 10×. (Right) Quantification of cell migrated and invaded cells in spheroid and adherent cells determined higher ability of mammospheres in migration and invasion. Data indicated the mean ± SD of three independent experiments. *P < 0.05; **P < 0.01 compared with parental cells.

In addition, we have analysed to assess if these cell lines differ in their metastatic function in vitro. Our results indicated that all three kinds of mammospheres showed a significant increase in invasion and migration in comparison with their monolayers, but triple negative breast cancer cells (MDA‐MB231 and MDA‐MB468) exhibited stronger invasive capacity as compared to luminal phenotype (MCF‐7) cells (Figure 3).

### 3.3 Mammospheres differentially expressed stemness‐ and metastasis‐related genes

**Figure 4:**
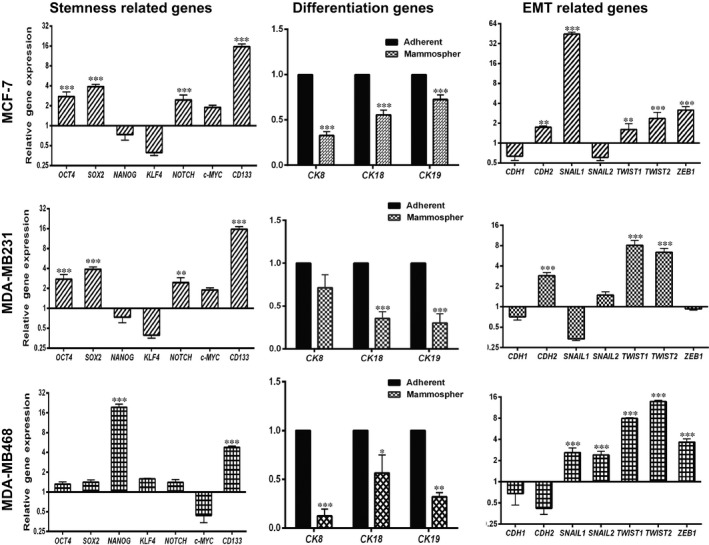
Mean values of fold change for stemness‐, differentiation‐, and metastasis‐related genes in MCF‐7, MDA‐MB231, and MDA‐MB468. Expression of stemness genes (left part), differentiation genes (middle part), and metastasis genes (right part) in mammosphere relative to adherent cells (control) determined by qRTPCR. β‐ACTIN mRNA was used as the housekeeping gene. Levels of gene expression for adherent culture (the black line has started from one). Each cell line represents n ≥ 3. Statistically significant difference was determined by paired *t* test with GraphPad Prism 6 software. Results were mean ± SEM. **P < 0.01; ***P < 0.001

Based on above results, we evaluated the expression pattern of some stemness‐related genes *OCT4, SOX2, NANOG, KLF4, NOTCH, c‐MYC*, and* CD133*, breast differentiation‐related genes *CK‐8, CK‐18*, and* CK‐19*, and EMT transcription factors *CDH1, CDH2, SNAIL1, SNAIL2, TWIST1, TWIST2*, and *ZEB1* in mammospheres and their counterpart’s adherent cells. Interestingly, the expression of *CD133* was up‐regulated in all three mammospheres and was dominant in MCF‐7 and MDA‐MB231 (15.66‐ and 9.97‐fold vs 4.76‐fold in MDA‐MB468 spheres, *P* < 0.0001). Moreover, mammospheres derived from MCF‐7 and MDA‐MB231 significantly overexpressed *SOX2 *(3.89‐fold, *P* < 0.0001 and 5.19‐fold, *P* < 0.002, respectively). However, MDA‐MB‐231 and MDA‐MB468 spheres just up‐regulated significantly the expression of *NANOG *(fold 3.39, *P*: 0.0061, fold: 19.39, *P < *0.0001) (Figure 4, right). By considering the overexpression of stemness‐related genes, almost all cytokeratin genes showed reduced expression in mammospheres (Figure 4, middle). Among EMT regulators, most of them were up‐regulated in mammospheres derived from MDA‐MB468 and MCF‐7. The MDA‐MB231 just overexpressed N‐cadherin (*CDH2)* and *TWIST1/2*. The expression of E‐cadherin (*CDH1*) was down‐regulated in most of mammospheres but was not significant (Figure 4, left).

### 3.4 Selection of MicroRNAs and prediction of their target genes and Gene ontology analysis

In order to select the miRNAs with the highest efficacy on self‐renewal and metastasis, we used the literature mining and integrated bioinformatics analysis by using of PubMed, Coremine, and GEO website. “Human breast cancer cell lines, CSC, self‐renewal, stemness, microRNA, metastasis, or EMT” were used as keywords. A total of 384 articles were yielded that some of them were excluded based on duplication of data (N = 18), no relation to stemness and EMT (N = 48), letters and reviews (N = 57). After full‐text reviewing of 266 remained articles, the most frequent miRNAs that regulated the stemness and metastasis genes were highlighted. A total of 143 miRNAs were found to be expressed in breast cancer cells; 88 of them were dysregulated in breast CSCs; 51 of them were up‐regulated and 37 of them were down‐regulated. Among them, 65 miRNAs regulated EMT and metastasis; 27 were underexpressed; and 38 were overexpressed (Supplementary Excel 1). Interestingly, 49 miRNAs were share in stemness and EMT group (Supplementary Excel 1). As a next step, we extracted target genes of 49 selected miRNAs by TargetScan^16^ and miRWalk.^17^, ^18^ To find the best miRNAs to target both stemness and metastasis genes, a threshold was defined (described in Section 2). As a result, eight miRNAs including miR‐200c‐3p, miR‐21‐5p, miR‐204‐5p, miR‐30c‐5p, miR‐34a‐5p, miR‐10b‐5p, mir‐520c‐3p, and mir‐373‐3p were found to target self‐renewal and EMT. The experimentally validated of target genes of each miRNAs has shown in Table 1.

**Table 1:** miRNAs involved in breast cancer metastasis and self‐renewal along with their target genes

microRNA	Metastasis genes	Stemness genes
miR‐10b	CDH1, CDH2, MYC, SNAIL1, SALL4, SMAD4, TWIST1, ZEB1	FAS, GLI1, KLF4, MYC, SOX2, TP53
miR‐21	CDH1, ETS1, FOSL1, GAS5, RELA, SNAIL1, STAT3, TGFB1, TGFB2, TWIST1, ZEB1	ELK1, FAS, GAS5, KLF4, MYC, NFKB1, NOTCH1, SOX2
miR‐30c	CDH1, DNMT1, HOXA1, MTA1, SNAI1, SNAI2, TWIST1, ZEB2	FAS, GLI1, KLF4, MYC, NOTCH1, SOX2, TP53, VIM3
miR‐34a	CDH1, FOX2, IL6, PLCE1, SMAD4, SNAI1, STAT3, ZEB1	CD44, FAS, GL1, KLF4, MYC, NANOG, NOTCH1, POU5F1, SOX2, TP53
miR‐200c	CFL2, FN1, MAPK9, MUC1, RHOA, ROCK2, SNAIL1, ZEB1/2	BMI1, KLF4, KRAS, NANOG, NOTCH1, SOX2, SP1, TP53
miR‐204	CDC42, CDH1, CDH2, NTRK2, SNAI1, SNAI2, STAT3, TWIST1	CD44, FOXC1, HOTTIP, MYC, NOTCH1, SOX2, STAT3, VIM1
miR‐373	BRF2, JAK2, LATS2, MYC, SNAIL1, TIMP2, TP53, VIM, ZEB1	CD44, TGFB1, TGFB2
miR‐520	HOXA, IRF2, SNAIL1	CD44, KLF4, NOTCH, SOX2

**Figure 5:**
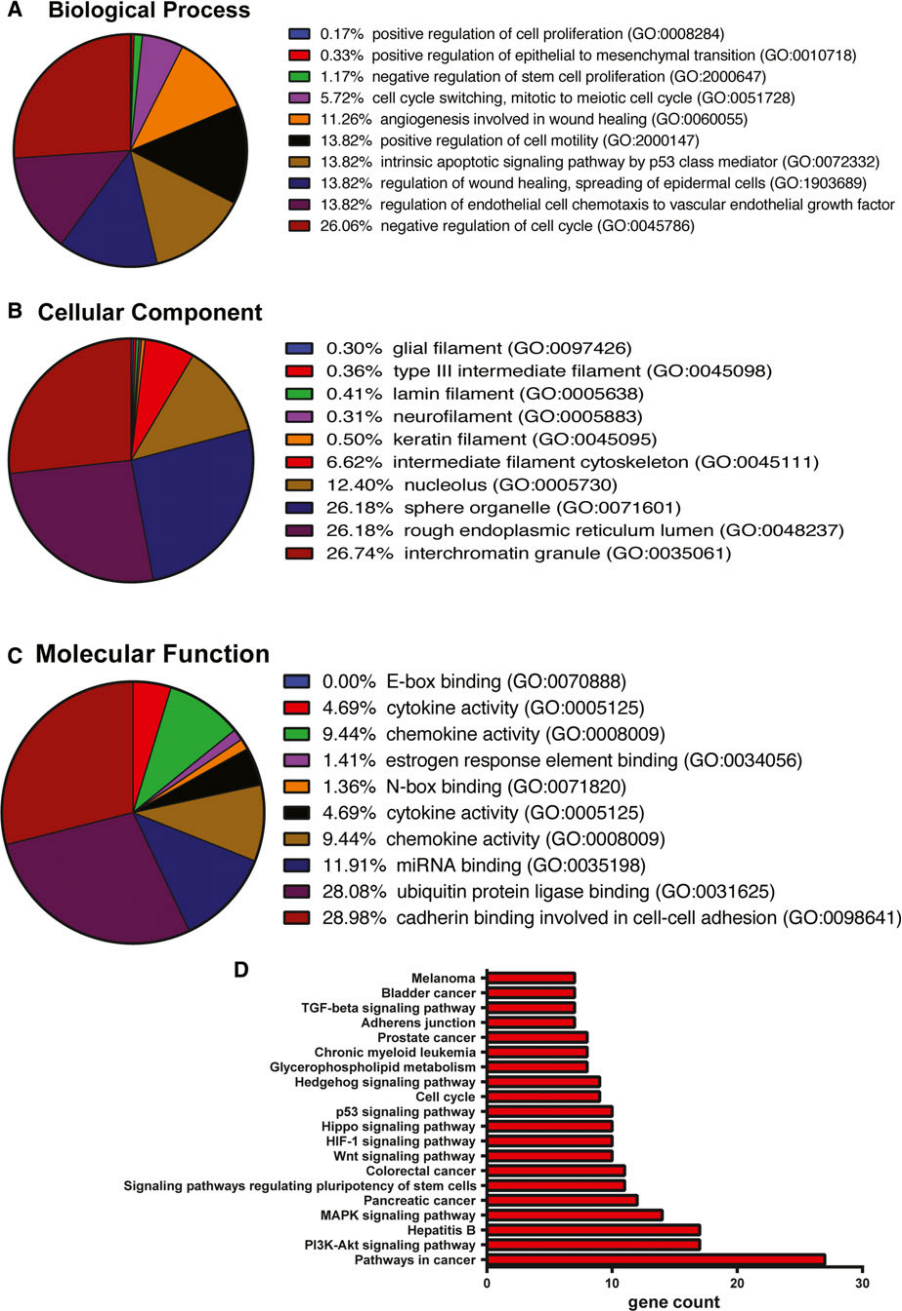
Gene Ontology (GO) and KEGG pathway analysis using Enrichr. The Stemness and EMT regulated genes from the differentially expressed miRNAs between mammospheres and adherent culture. Only the top ten enriched GO terms are represented in the respective pie charts. The enriched Kyoto Encyclopedia of Genes and Genomes (KEGG) pathways of the 35 selected target genes of 8 microRNAs

In order to gain a better understanding of the specific biological functions of eight selected miRNAs, their target genes were identified from three database: miRTarbase, TargetScan,^16^ and miRWalk.^17^, ^18^ We arrived to the set of 34 target genes in three steps: first, we establish the set of genes that had overlapped among the selected miRNAs. As a second step, the list of target genes was registered in the GO annotation data set for biological process, molecular function, and cellular components using Enricher. Third, the list was sorted based on *P*‐value, number of genes, and known functions for mRNAs with self‐renewal, stemness, invasion, or migration. The most significantly enriched genes were involved in biological process of the cell‐cell adhesion, stem cell proliferation process, cell cycle, and EMT process (Figure 5A). In cellular component, most of the genes were belonged to the nucleolus and cytoplasmic organelles (Figure 5B). In molecular function, the term with the lowest *P*‐value was E‐box binding, DNA binding, N‐box binding, cadherin binding involved in cell‐cell adhesion, and miRNA binding (Figure 5C). Finally, KEGG pathway analysis showed similar results, with the number of genes involved in the cell adhesion molecules (CAMs), pathways in cancer, MAPK signalling pathway, Wnt signalling pathway, Hedgehog signalling pathway, Hippo signalling pathway, transforming growth factor β (TGF‐β) signalling pathway, signalling pathways regulating pluripotency of stem cells, p53 signalling pathway, and cell cycle (Figure 5D). This confirmed the network of miRNA‐mRNA interactions for selected eight miRNAs in regulation of self‐renewal and EMT process (Figure S2).

### 3.5 Mammospheres differentially expressed MicroRNAs targeting both stemness and EMT pathways

**Figure 6:**
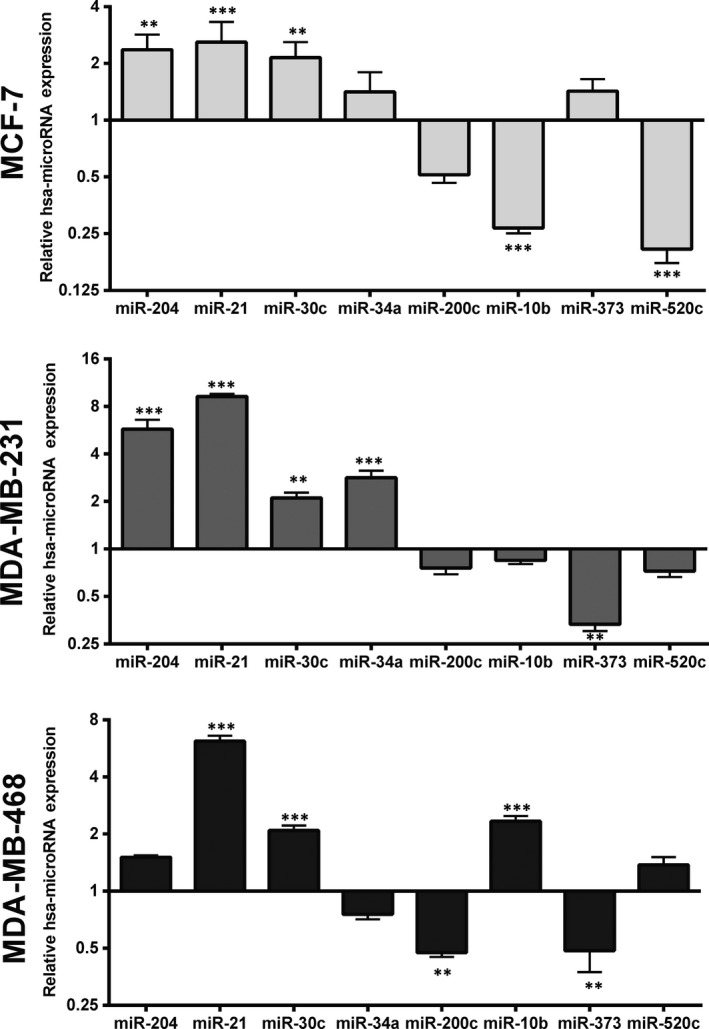
Expression (mean ± SD) of miR‐204, miR‐21, miR‐30c, miR‐34a, miR‐200c, miR‐10b, miR‐373, and miR‐520c between mammospheres derive of MCF‐7, MDA‐MB231, and MDA‐MB468 vs adherent culture (as control) determined by qRT‐PCR. The expression of each miRNA was normalized to the levels of u6. Each cell lines represent n ≥ 3. **P < 0.01; ***P < 0.001

Eight above‐selected miRNAs with potential to target self‐renewal and EMT pathways were evaluated in mammospheres derived from different cell lines. Interestingly, as shown in Figure 6, miR‐204, miR‐21, and miR‐30c were overexpressed in all spheroid types; however, miR‐204 was not significant in mammospheres of MDA‐MB468 (*P*: 0.1936). Although each groups differentially expressed all eight miRNAs, some similarities were also observed. miR‐10b, miR‐34a, and miR‐520c were resembled similar in MCF‐7 and MDA‐MB231 spheroids. The similarity between MCF‐7 and MDA‐MB468 was in the expression of miR‐30c and miR‐200c, and finally, the expression pattern of miR‐373 was similar in both MDA‐MB231 and MDA‐MB468 spheres (Figure 6). These similarities or verities may be the results of expression pattern of stemness‐ and EMT‐related genes in derived mammospheres. As shown in Table 2, most of miRNAs in mammospheres derived from MCF‐7, had correlation with OCT4 (mostly negative), and were positively correlated to *SOX2*, *KLF4*, *c‐MYC*, and *CD133* genes. Similar to MCF‐7‐mammospheres, miR‐200c of MDA‐MB231 mammospheres and miR‐34a of MDA‐MB468 mammospheres had negative and positive correlation with *OCT4* and *c‐MYC* respectively (Table 2). Among miRNAs, miR‐10b and miR‐21 had negative correlation with CK8. However, miR‐520c was positively and miR‐21 was negatively correlated with CK18 expression (Table 2). The most miRNA‐mRNA correlation related to metastasis was belonging to mammospheres derived from MCF‐7 and MDA‐MB468. In overall, we suggested miR‐21 and miR‐200c with higher connection to *OCT4, SOX2*, and *KLF4* and with lower connection with miR‐34a (correlation with *c‐MYC and CD133*) and miR‐204 (with *OCT4* and *c‐MYC*) on stemness regulation. Furthermore, miR‐204 showed more strong correlation with EMT‐related genes (*CDH1, CDH2, TWIST2*) and then miR‐10b (*ZEB1, SNAIL2*) and miR‐34a (*CDH1, CDH2*) were relevant in above pathway (Table 2). To identify prognosis‐related miRNAs, we first used the bivariate correlation analysis to evaluate the associations between the expression level of each of the eight differentially expressed miRNAs with EMT‐ and stemness‐related genes and found that eight miRNAs (Table 2) were significantly associated with the genes expression (*P* < 0.05).

**Table 2:** Correlation coefficient of miRNA‐mRNA related to stemness, differentiation, and metastasis pathway

Stemness genes							
	OCT4	SOX2	NANOG	KLF4	NOTCH	cMYC	CD133
MCF‐7	miR21 (−0.975**) miR200c (−0.823*) miR204 (−0.897*) miR373 (0.982**)	miR21 (0.845*) miR200c (0.810*) miR373 (−0.837*)	NS	miR10b (0.935**)	NS	miR30c (0.878*) miR34a (0.922**)	miR30c (0.992**) miR34a (0.856*) miR520c (0.861*)
MDA‐MB‐231	miR200c (−0.899*)	NS	NS	NS	NS	miR204 (0.886*)	NS
MDA‐MB‐468	NS	NS	miR373 (−0.844*)	miR21 (−0.805*) miR200c (0.802*)	NS	miR34a (0.910*)	NS

Metastasis genes							
	CDH1	CDH2	SNAIL1	SNAIL2	TWIST1	TWIST2	ZEB1
MCF‐7	miR204 (−0.862*)	miR204 (0.804*)	miR30c (0.963**) miR520c (0.835*)	miR10b (0.877*)	miR520c (−0.823*)	NS	
MDA‐MB‐231	miR34a (0.943**)	miR34a (−0.829*)	NS	NS	NS	miR204 (−0.829*)	miR10b (−0.829*)
marMDA‐MB‐468	NS	NS	miR204 (−0.822*) miR373 (0.964**)	NS	NS	miR204 (−0.979**) miR373 (0.816*)	miR10b (−0.953**) miR21 (−0.857*)

Differentiation genes				
	CK8	CK18	CK19	
MCF‐7	NS	miR520c (0.828*)	NS	
MDA‐MB‐231	miR10b (−0.829*)	miR21 (−0.886*)	NS	
marMDA‐MB‐468	miR21 (−0.857*)	NS	NS	

* *P* < 0.05.

** *P* < 0.01.

### 3.6 miRNAs vs pathways heat maps and the survival implication for selected miRNAs

**Figure 7:**
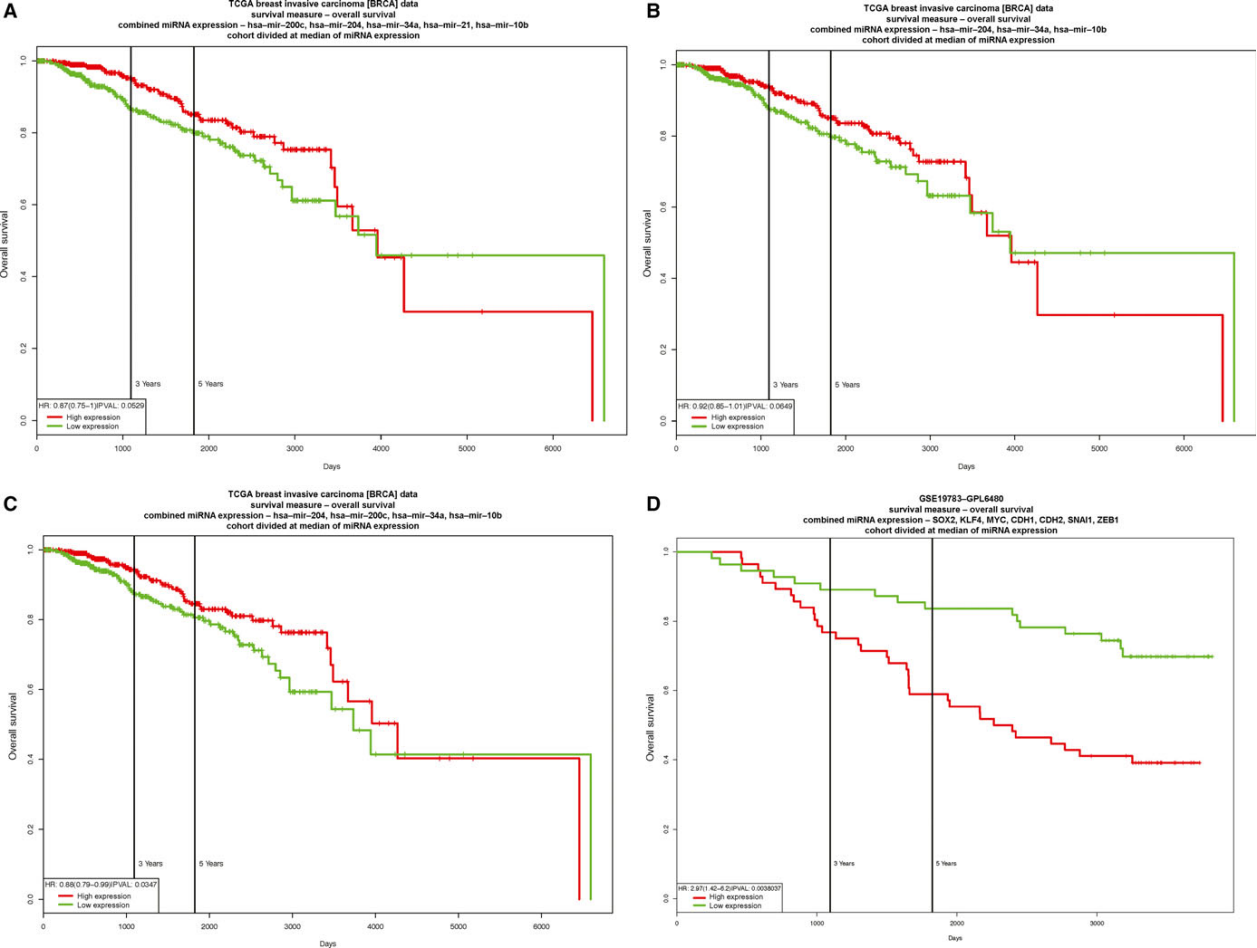
Diagnostic plots created with PROGmiR for published signatures in breast invasive carcinoma (BRCA). (A) Kaplan‐Meier survival curve analysis for overall survival of breast invasive carcinoma patients using the five‐miRNA regulate stemness genes. (B) Prognostic evaluation of the three miRNAs that act as metastatic regulator genes were associated with overall survival in breast cancer patients. (C) The miRNAs that regulate both of stemness and metastasis genes. (D) The stemness‐ and metastasis‐related genes in the patients were stratified into a high‐risk group and a low‐risk group according to median of each miRNA

Based on our correlation results, we have divided miRNAs into three groups; miRNAs that regulate EMT‐related genes including miR‐204, miR‐10b, and miR‐34a; miRNAs which regulate stemness and differentiation related genes including miR‐204, miR‐21, miR‐200c, miR‐34a, and miR‐10b; and finally, miRNAs that regulate both self‐renewal and EMT including miR‐204, miR‐200c, miR‐34a, and miR‐10b. In order to recognize the role of miRNAs in cancer development, the DIANA‐mirPath analysis of the selected miRNAs in each group was performed. As shown in Figure 6, most of miRNAs significantly modulated in most of cancers including glioma, pancreatic, bladder, non‐long carcinoma, prostate, thyroid cancers, and also p53 pathway, cell cycle, pyrimidine metabolism, cell cycle, and DNA replication. Using PROGmiR made us able to create a significant diagnostic plot between the expression level of each set of miRNAs and patients overall survival. As shown in Figures S3 and S7, the expression levels of most miRNAs and also combination of those miRNAs in group 1 (as targeting for EMT) and group two (as targeting for stemness) had no significant effect on the survival rate of breast cancer patients (Figure 7A,B). However, combination of miR‐204, miR‐200c, miR‐34a, and miR‐10b significantly reduced the survival rate of breast invasive carcinoma (*P*: 0.03, Figure 7C). Moreover, their main targets in self‐renewal and EMT pathways also significantly reduced the overall survival of breast invasive carcinoma patients (*P*: 0.0038, Figure 7D).

## 4 DISCUSSION

The present study described the evaluation of the expression of miRNAs that target both stemness‐ and EMT‐related genes in the three‐dimensional (3D) spheroid‐enriched BCSCs model. For these, first we focused on the establishment of define medium and coating layer to form mammosphere‐enriched BCSCs. There are different methods for mammospheres cultivation^24^ that usually apply poly‐2‐hydroxyethyl‐ methacrylate (p‐HEMA),^25^ agaros,^26^ agar,^27^ and matrigel^28^ to provide low attachment surface. However, the efficiencies of each one in production of spheres are still unclear. Moreover, the type of culture media may affect the spheroid‐enriched BCSCs. Our results determined that the mammospheres cultured in agar‐coated plate and in the presence of DMEM were compact and larger in size with higher ability of colony and sphere formation efficiency. Therefore, they may have higher level of BCSCs. Interestingly, all mamosphere types derived from MCF‐7, MDA‐MB231, and MDA‐MB468 were more capable to form higher spheres and holoclones compared to their parental cells in agar‐coated plate and in the presence of DMEM. Among three types of cells, mammospheres derived from MCF‐7 overexpressed most of stemness‐related genes (*OCT4, SOX2, NOTCH, and CD133*), then MDA‐MB231 mammospheres up‐regulated significantly *SOX2, NOTCH, KLF4*, and *CD133*, while *c‐MYC* was underexpressed. In mammospheres derived from MDA‐MB468, just *NOTCH* and *CD133* were overexpressed and *c‐MYC* was underexpressed. All these changes were associated with a reduction in differential related genes. Most of evidences have suggested that the cancer progression is associated with CSCs acquisition of the EMT phenotype, which is responsible for increased cell motility and invasion.^29^ All three types of mammospheres revealed greater ability to migrate and invade in vitro, which was associated with increasing in mRNA level of EMT transcription factors. EMT factors were up‐regulated most in MDA‐MB‐468‐derived mammospheres. Along with other studies, we also suggested the greater potential of MCF‐7 and MDA‐MB231 spheres in repopulation;^30^, ^31^ however, spheres derived from MDA‐MB‐468 were more potent to induce metastasis.^32^

As a next step, by using of literature and data mining, we highlighted eight miRNAs including “miR‐200c, miR‐21, miR‐204, miR‐30c, miR‐34a, miR‐10b, miR‐520c, and miR‐373” with ability to target both stemness and EMT pathways in breast cancer. Interestingly, the mammospheres derived from MCF‐7 and MDA‐MB‐231 resembled more similarity in stemness‐related genes expression, but MDA‐MB‐231‐mammospheres had lower potential of clonogenicity which may be due to the lack of changes in the expression of miR‐10b, ‐520. Nevertheless, more detailed studies are needed.

Regardless of all variety micro‐RNA expression, the similarity was found in expression of miR‐204, miR‐21, and miR‐30c (all were up‐regulated) in all spheroid types, while miR‐200c was reduced. Remarkably, all four miRNAs had highest correlation with *OCT4, SOX2, cMYC*, and *CD133 *(belong to stemness‐related genes), especially in MCF‐7‐mammospheres. The correlation of miR‐204 with *CDH1, CDH2, SNAIL1, TWIST2*, and *ZEB1* (of EMT factors) was dominant in all types of mammospheres. Among all mentioned miRNAs, the role of miR‐21 and miR‐200c is more defined in acquisition of CSC signatures^33^ and regulation of EMT programme^34^ in various kinds of human cancers including breast cancer.^33^, ^35^, ^36^ The expression of miR‐200c in the breast CSCs inhibits the proliferation of breast cancer cells through the regulation of metastasis genes including *ZEB1* and *SNAIL1*,^37^, ^38^ similarly stemness gene such as *NOTCH1*.^39^, ^40^ The down‐regulation of miR‐200c was speculated to be the reason of high radiation tolerance in tumour cells.^41^ The expression of miR‐30c also is associated with the acquisition of EMT phenotype by directly targeting of *ZEB1, CDH1*, and *SNAIL1* in breast tumours^42^, ^43^ and has role in self‐renewal by directly targeting of *NOTCH1, c‐MYC*, and *CD44.* 40

Although the role of miR‐200c, miR‐21, and miR‐30c in regulation of CSCs is well defined, the role of miR‐204 in cancers and CSCs is controversial. Most of the studies have reported miR‐204 as a tumour suppressor gene, and its lower expression is significantly associated with a more aggressive tumour phenotype in breast cancer,^44^ with poor clinical outcome of acute myeloid leukaemia patients,^45^ poor survival in colorectal cancer patients,^46^ and inhibit migration and invasion of cervical cancer.^47^ Several studies also suggested miR‐204 as an onco‐miR in breast cancer^48^ and its overexpression was also shown to increase the migration, invasion, and metastasis of breast cancer MCF‐7, MDA‐MB231, and MDA‐MB468 cells.^48^ It may play a regulatory function in stem cells through targeting the CD44 and NOTCH.^49^, ^50^ Meanwhile, Wang et al reported the inhibitory effect of miR‐204 in the self‐renewal of breast cancer cells.^51^

Our results determined that, although miR‐204 may act as onc‐miR in BCSCs and along with other miRNAs, it has an effective role in determination of BCSCs final fate.

**Figure 8:**
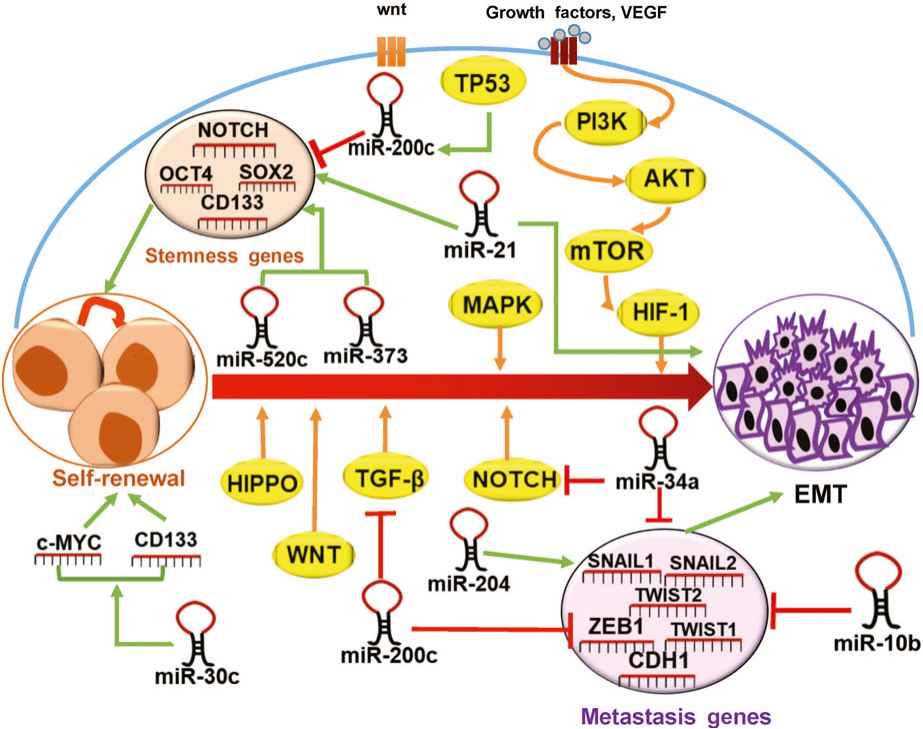
Schematic illustration of selected miRNAs on individual components of signalling pathways related to stemness and metastasis

miR‐34a and miR‐10b target/regulate some of stemness genes and EMT factors but with lower correlation to both pathways. The role of both miRNAs in BCSCs has been reported previously in several studies.^52^, ^53^, ^54^ The pathway analysis revealed that these genes were significantly related to the “CAMs,” “pathways in cancer,” “MAPK signalling pathway,” “Wnt signalling pathway,” “Hedgehog signalling pathway,” “Hippo signalling pathway,” “TGF‐β signalling pathway,” “Signalling pathways regulating pluripotency of stem cells,” “p53 signalling pathway,” “ABC transporters,” and “Cell cycle.” All these pathways have been demonstrated to be linked to various cellular activities including proliferation, migration, invasion, formation of multicellular spheroid, regulation of oestrogen receptor signalling, cancer progression, metastasis, self‐renewal in cancer and CSCs, maintenance of EMT and stemness, and breast cancer chemoresistance. Interestingly, TGF‐β induces miR‐21^55^and miR‐204^56^ expression, represses c‐MYC, a transcription factor that promotes cell proliferation, and inhibits cell differentiation.^57^ GO analysis also showed noticeable changes in cellular components of the spheroids, which gives the spheroids higher cell proliferation and cell migration characteristics. Actually, deregulation of miR‐204, miR‐200c, miR‐34a, and miR‐10b simultaneously could significantly reduce the survival rate of breast invasive carcinoma via up‐regulation of *OCT4, SOX2, KLF4, c‐MYC, NOTCH1, SNAI1, ZEB1, *and *CDH2 *and down‐regulation of *CDH1 *(Figure 8).

## 5 CONCLUSIONS

To the best of our knowledge, this is the first demonstration of the involvement of a combination of specific miRNAs in the coordinated regulation of BCSC proliferation, EMT, and differentiation. We suggested here that the miR‐204, miR‐200c, miR‐34a, and miR‐10b form a core regulatory network for induction of self‐renewal and EMT in BSCs and affect the survival rate of breast invasive carcinoma patients. However, further studies are needed to elucidate these potential of miRNAs in CSCs fate determination. Moreover, they can be considered as ideal diagnostic marker in blood and could be targeted for breast cancer therapy.

## CONFLICT OF INTEREST

The authors confirm that there are no conflicts of interest.
