# Supplementary material for: An integrated analysis to predict micro‐RNAs targeting both stemness and metastasis in breast cancer stem cells
Source: J Cell Mol Med. 2019 Feb 1;23(4):2442–56. doi: 10.1111/jcmm.14090 (PMC6433858; doi:10.1111/jcmm.14090)
Supplement: Supplementary file 1 [file JCMM-23-2442-s001.docx]

**Supplementary Table 1:** Primer sequences were used for qRT-PCR.

| **Primer** | **Sequence** | **Length(bp)** | **Accession number** |
| --- | --- | --- | --- |
| **SOX2** | **F: 5' GGG AAA TGG AAG GGG TGC AAA AGA GG 3'**  **R: 5' TTG CGT GAG TGT GGA TGG GAT TGG TG 3'** | **151** | **NM_003106.3** |
| **KLF4** | **F: 5' ATTACCAAGAGCTCATGCCA 3'**  **R: 5' CCTTGAGATGGGAACTCTTTG 3'** | **150** | **NM_004235.4** |
| **NANOG** | **F: 5' AAAGAATCTTCACCTATGCC 3'**  **R: 5' GAAGGAAGAGGAGAGACAGT 3'** | **110** | **NM_02abi4865.2** |
| **OCT4** | **F: 5' CTGGGTTGATCCTCGGACCT 3'**  **R: 5' CACAGAACTCATACGGCGGG 3'** | **128** | **NM_002701.4** |
| **c-MYC** | **F: 5' ACACATCAGCACAACTACG 3'**  **R: 5' CGCCTCTTGACATTCTCC 3'** | **140** | **NM_002467** |
| **NOTCH** | **F: 5' CAGACCCACACCCAGTA 3'**  **R: 5' GGCAACGTCAACACCTT 3'** | **114** | **NM_017617** |
| **CD133** | **F: 5' GCA TCC ATC AAG TGA AAC GT 3'**  **R: 5' GGT TTG GCG TTG TAC TCT GT 3'** | **199** | **NM_001145852.1** |
| **CK8** | **F: 5' CAGATCAAGACCCTCAACAAC 3'**  **R: 5' CACTTGGTCTCCAGCATCTT 3'** | **89** | **NM_001256293.1** |
| **CK18** | **F: 5' GCG AGG ACT TTA ATC TTG GTG 3'**  **R: 5' CTT TGG TGT CAT TGG TCT CAG 3'** | **120** | **NM_199187.1** |
| **CK19** | **F: 5' GCGACTACAGCCACTACTACA 3'**  **R: 5' TGGTTCGGAAGTCATCTGC 3'** | **129** | **NM_002276.4** |
| **CDH1** | **F: 5' CAG GAG TCA TCA GTG TGG T 3' R: 5' GGA GGA TTA TCG TTG GTG TCA G 3'** | **150** | **NM_004360.3** |
| **CDH2** | **F: 5' GCCCAAGACAAAGAGACCC 3'**  **R: 5' CTGCTGACTCCTTCACTGAC 3'** | **93** | **NM_001792.3** |
| **SNAIL1** | **F:5' CCAGAGTTTACCTTCCAGCA 3'**  **R:5'GATGAGCATTGGCAGCGA 3'** | **101** | **NM_005985.3** |
| **SNAIL2** | **F:5' AACTACAGCGAACTGGACAC 3'**  **R:5'GGATCTCTGGTTGTGGTATGAC 3'** | **90** | **NM_003068.3** |
| **TWIST1** | **F: 5' CCA GGT ACA TCG ACT TCC TC 3'**  **R: 5' TCG TGA GCC ACA TAG CTG 3'** | **85** | **NM_000474.3** |
| **TWIST2** | **F: 5' GAG GAGATT CTG AAT GAT GCT 3'**  **R: 5' GTC TTG ACC AAA CAC TGC C 3'** | **87** | **NM_057179.2** |
| **ZEB1** | **F: 5' GAGGATGACACAGGAAAGGA 3'**  **R: 5' CAGCAGTGTCTTGTTGTTGT 3'** | **163** | **NM_001128128.2** |
| **β-actin** | **F: 5' TCCCTGGAGAAGAGCTACG 3'**  **R: 5' GTAGTTTCGTGGATGCCACA 3'** | **131** | **NM_ 001101.3** |

**Supplementary Figure 1** Flowchart of systematic analysis based on literature and data mining to select miRNAs that target both stemness and EMT pathways.

**Supplementary Figure 2** Network of differentially expressed miRNAs-target genes. The color of the genes indicates the amount of interactions they are involved in. Orange genes are targeted by three or more microRNAs, genes that are targeted by two microRNAs are colored Blue. The interactions shown here are all experimentally validated. Interactions with strong experimental evidence are depicted by green edges. The EMT related genes are colored red edges and the Stemness related genes are colored green edges.

**Supplementary Figure 3** Diagnostic Plots of selected micro-RNAs created by PROGmiR for published signatures in Breast invasive carcinoma [BRCA].
